# Alveolar Bone Loss at Maxillary and Mandibular Permanent First Molars in Periodontitis Patients: A Descriptive Cross-sectional Study

**DOI:** 10.31729/jnma.9133

**Published:** 2025-07-31

**Authors:** Harish Kumar Shah, Rajesh Shah, Sujaya Gupta, Harendra Mohan Singh, Asdaq Hussain, Shakeel Ahmad, Abanish Singh

**Affiliations:** 1 Department of Periodontology and Oral Implantology, National Medical College Teaching Hospital, Birgunj, Parsa, Nepal; 2 Department of Periodontics and Oral Implantology, Kathmandu Medical College Teaching Hospital, Duwakot, Bhaktapur, Nepal; 3 Department of Conservative Dentistry and Endodontics, National Medical College Teaching Hospital, Birgunj, Parsa, Nepal; 4 Department of Orthodontics, National Medical College Teaching Hospital, Birgunj, Parsa, Nepal; 5 Australian Research Centre for Population Oral Health, Adelaide Dental School, The University of Adelaide, Australia.

**Keywords:** *alveolar bone loss*, *panoramic radiograph*, *periodontitis*, *tobacco smoking*, *toothbrushing*

## Abstract

**Introduction:**

Periodontitis is an inflammatory condition of periodontium that results in alveolar bone loss. Panoramic radiograph is one of the methods used to evaluate alveolar bone loss. However, in Nepal data quantifying the extent and site-specific pattern of alveolar bone loss using panoramic radiograph are still lacking. The objective of this study was to determine the amount of alveolar bone loss at the mesial and distal sites of maxillary and mandibular permanent first molars.

**Methods:**

This descriptive cross-sectional study was conducted in the Department of Periodontology and Oral Implantology at National Medical College from 15 July 2023 to 15 March 2024 among 190 periodontitis patients using convenience sampling method after institutional ethical clearance. Alveolar bone loss at mesial and distal sites was measured in millimeters from cementoenamel junction to alveolar crest on a digital orthopantomogram. Data was entered in Microsoft Excel Sheet and descriptive analysis was done. The findings are presented as frequencies, percentages, means, and standard deviations. The point estimate was calculated at a 95% confidence.

**Results:**

In patients with periodontitis, the alveolar bone losses at mesial and distal sites of the maxillary first molars were 4.27±1.07 (95% CI: 4.11-4.41) mm and 4.45±1.12 (95% CI: 4.29-4.62) mm. For mandibular first molars the corresponding values were 3.69±0.92 (95% CI: 3.55-3.82) mm and 3.87±1.03 (95% CI: 3.72-4.01) mm. The mean alveolar bone loss was found to be higher among smokers, older age groups, and those who brushed their teeth only once a day.

**Conclusion:**

The alveolar bone loss was greater in the maxillary first molar than mandibular first molar.

## INTRODUCTION

Periodonitis is an infectious and inflammatory disease of the periodontium that results in alveolar bone loss (ABL).^1^ It is one of the most prevalent oral diseases in Nepal.^2^ Various methods have been used to measure ABL, among which panoramic radiographs are considered valid and reproducible when measuring the distance from cementoenamel junction (CEJ) to alveolar crest.^3^ Angular defects have been found most frequently in the upper and lower molars.^4,5^

The ABL evaluation is one of the crucial factors for determining the prognosis, treatment plan, and outcome of periodontal therapy in periodontitis patients. In many cases, the decision to retain or extract a tooth often depends on the extent of ABL. This study was also first of its kind in the Nepalese population.

The aim of present study was to determine the amount of ABL at the mesial and distal sites of the maxillary and mandibular permanent first molars using panoramic radiograph.

## METHODS

This descriptive cross-sectional study was conducted among 190 patients diagnosed with periodontitis according to 2017 classification of periodontal and periimplant diseases and conditions,^1^ who visited the department of Periodontology and Oral Implantology, National Medical College, Birgunj-15, Parsa, Nepal, from July 15, 2023 to March 15, 2024 and met the study inclusion and exclusion criteria. Ethical approval was obtained from the Institutional Review Committee of the same institute (Reference number: F-NMC/657/079-080) prior to commencement of the study. Informed written consent was obtained from all participants to include them in the study.

The inclusion criteria were, patients diagnosed with periodontitis, aged between 20 years and 60 years, either male or female, presence of both maxillary and mandibular permanent first molars, and at least 20 permanent teeth present in both jaws. The exclusion criteria were, absence of either mandibular or maxillary permanent first molars, distorted, overlapped, or unclear radiographic images, or inability to identify the interproximal projection of the CEJ.

A convenience sampling method was employed to collect the required sample for this study. For sample size calculation, a study done by Mhatre et al.^6^ was considered. According to them, mean alveolar bone loss occurring on the mesial site was 5.23±2.315 mm at a mandibular first molar, with relative permissible error (e) =6.5% of mean. Using the standard formula:

n= Z^2^ × σ^2^ / e^2^

 = 3.84 × 5.359225/0.115566

 ≈ 178

Where,

 Z = 1.96 for 95% confidence interval

 σ = standard deviation

 e = relative margin of error = 6.5%.

To account for non-responses, incomplete data, dropouts, or to make it more reliable, we included an additional margin of 6.5% of obtained sample size, rounding off the sample size to 190.

The sample was categorized based on several variables. According to site of involvement (only one finding from either right or left of maxillary and mandibular first molar site was recorded which met inclusion and exclusion criteria), four groups were formed: (1) radiographic alveolar bone loss at the mesial site in either of right or left of the maxillary permanent first molar; (2) radiographic alveolar bone loss at distal site in either of right or left of the maxillary permanent first molar; (3) radiographic alveolar bone loss at mesial site in either of right or left of the mandibular permanent first molar; and (4) radiographic alveolar bone loss at distal site in either of right or left of the mandibular permanent first molar. Based on age groups, participants were divided into four groups: 20-29 years, 30-39 years, 40-49 years, and 50-60 years. According to gender, categorized into both males and females. Brushing frequency was categorized as once daily or twice daily. Lastly, participants were also grouped based on smoking status into smokers and non-smokers.

The demographic profile of the patients was recorded followed by brushing frequency (once or twice a daily, smoking status: smoker (smoking cigarette) or non-smoker (not smoking cigarette), along with clinical examination with University of North Carolina-15 periodontal probe to diagnose periodontitis. The orthopantomogram (OPG) image was taken using PaX-i 2D Imaging System (VATECH Global, Korea; Model: PCH-2500) having power unit 100-120/200-240 V, 50/60 Hz, 2.0 kVA max 170 VA nominal. The images were analyzed on an 18.5-inch Dell monitor (Model: E1912H; resolution: 1280 × 1024 pixels) using EzDent-i software (VATECH Global, Korea). Alveolar bone loss was measured in millimeters as the distance from the CEJ to the alveolar crest by a single examiner (HKS).

Data were entered in MS Excel sheet 2019 and descriptive analysis done. The findings have been presented as frequency, percentage, mean, and standard deviation. The point estimate was calculated at a 95% confidence.

## RESULTS

The alveolar bone losses were measured at mesial and distal sites of maxillary and mandibular permanent first molars in 190 periodontitis patients. Out of 190 patients, 106 (55.78%) were male and 84 (44.21%) were female. Similarly, 43 (22.63%) were smokers, and the rest 147 (77.36%) were non-smokers. Additionally, patients who brushed their teeth once a day were 155(81.57%) and those who brushed twice a day were 35 (18.42%), (Table 1).

**Table 1:** Demographics of study participants (N=190).

Age category (years)	n(%)
20-29	47(24.74)
30-39	42(22.11)
40-49	50(26.31)
50-59	51(26.84)
Sex	
Male	106(55.78)
Female	84(44.22)
Marital status	
Married	165(86.84)
Unmarried	2 (13.16)
Smoking status	
Yes	43(22.63)
No	14(77.37)
Brushing frequency	
Once a day	15(81.58)
Twice a day	35(18.42)

**Table 2:** Descriptive statistics of-alveolar bone loss at mesial and distal sites of maxillary and mandibular first molars (n=190).

	Mean±SD (mm)
Maxillary first molar mesial	4.27±1.07
Maxillary first molar distal	4.45±1.12
Mandibular first molar mesial	3.69±0.92
Mandibular first molar distal	3.87±1.03

**Table 3:** Alveolar bone loss comparison between gender, smoking status, and brushing frequency (n=190)

Group	Maxillary first molar mesial	Maxillary first molar distal	Mandibular first molar mesial	Mandibular first molar distal
Gender				
Male (106)	4.37±1.09	4.57±1.16	3.75±0.99	3.94±1.12
Female (84)	4.14±1.02	4.31±1.06	3.61±0.81	3.77±0.90
Smoking status				
Yes (43)	4.93±1.05	5.13±1.08	4.11±0.95	4.38±1.11
No (147)	4.07±0.99	4.26±1.06	3.56±0.87	3.72±0.95
Brushing frequency				
Once a day (155)	4.36±1.03	4.56±1.11	3.77±0.92	3.95±1.03
Twice a day (35)	3.83±1.11	3.98±1.08	3.31±0.82	3.51±0.97

**Table 4:** Comparison of the mean bone loss among different age groups (n=190).

	20-29 years (mm)	30-39 years (mm)	40-49 years (mm)	50-59 years (mm)
Maxillary first molar mesial	3.10±0.58	3.73±0.73	4.74±0.68	5.32±0.51
Maxillary first molar distal	3.27±0.62	3.90±0.77	4.91±0.78	5.55±0.58
Mandibular first molar mesial	2.76±0.57	3.39±0.71	3.96±0.67	4.52±0.62
Mandibular first molar distal	2.91±0.65	3.59±0.81	4.16±0.91	4.69±0.75

The alveolar bone losses in the maxillary first molars at the mesial and distal sites were 4.27±1.07 mm (95% CI: 4.11-4.41) mm and 4.45±1.12 mm (95% CI: 4.29-4.62) mm, respectively and in the mandibular first molars mesial and distal sites were 3.69±0.92 mm (95% CI: 3.55-3.82) mm and 3.87±1.03 mm (95% CI: 3.72-4.01) mm, respectively (Table 2).

The alveolar bone losses in the maxillary first molars at the mesial site in smokers and non-smokers were 4.93±1.05 mm and 4.07±0.99 mm, respectively. Similarly, the alveolar bone losses in the maxillary first molars at the distal site in smokers and nonsmokers were 4.56±1.11 mm and 3.98±1.08 mm, respectively. Moreover, alveolar bone losses in the mandibular first molars at the mesial site in smokers and non-smokers were 4.11±0.95 mm and 3.56±0.87 mm, respectively. Additionally, alveolar bone losses in the mandibular first molars at the distal site in smokers and non-smokers were 4.38±1.11 mm and 3.72±0.95 mm, respectively (Table 3).

## DISCUSSION

Periodontitis is a multifactorial inflammatory disease associated with plaque biofilms, leading to progressive loss of attachment and alveolar bone. There are several methods for assessing alveolar bone loss, among which radiographic assessment is the most commonly used, as it is non-invasive and well accepted by patients. Among radiographic techniques, panoramic radiographs are widely used to evaluate the extent of alveolar bone loss, as they provide a comprehensive view of both the maxillary and mandibular arches. Detection of bone loss using orthopantomogram (OPG) is a practical method for screening alveolar bone loss. The assessment of alveolar bone loss through OPG plays an important role in determining the severity, prognosis, and appropriate treatment planning for patients with periodontitis. In this study, first molars were selected as they are the earliest erupting permanent teeth, remain longest in the oral cavity, and are highly susceptible to plaque-induced alveolar bone loss. Prior studies have also reported frequent angular defects in these sites, making them clinically significant for periodontal assessment.^3,4^

In the present study, alveolar bone loss was measured from the CEJ to the crest of the alveolar bone in millimeters, similar to the methods used by Mhatre et al., Bjorn et al., Schei et al., and Suomi et al.^6-9^ This direct method of measuring bone loss from the CEJ to the alveolar crest allows for a precise and reproducible evaluation of actual alveolar bone loss. ^3^ This approach is consistent with evaluations done using both panoramic and intraoral radiographs.^10^ The study found that alveolar bone loss in males was higher than in females, aligning with the findings of Mhatre et al.^6^ However, this result contrasts with the study by Van der Velden (1989), which reported a female to male bone loss ratio of 1.3 to 1.9 for aggressive periodontitis.^11^ Similarly, the present findings differ from those of Zardawi et al., who found that females exhibited non-significantly higher bone loss than males.^12^ The severity of periodontitis increases in the presence of smoking habits. In this study, alveolar bone loss was found to be higher among smokers compared to non-smokers, which is consistent with the findings of studies conducted by Zhang et al., Natto et al., Amaranath et al., and Helmi et al.^13-16^ These discrepancies could be attributed to differences in the study populations, including factors such as prevalence of smoking, oral hygiene practices, and the higher occurrence of periodontitis among males, which is often associated with increased alveolar bone loss.^16^

Dental plaque is the most common cause of periodontitis, and its formation is closely associated with oral hygiene practices. Poor oral hygiene contributes to increased plaque accumulation, leading to a greater severity of periodontitis and alveolar bone loss. In the present study, individuals who brushed their teeth only once a day exhibited greater alveolar bone loss compared to those who brushed twice daily. This finding aligns with studies by Schei et al. and Zimmermann et al.^8,17^

Periodontitis was observed across all age groups; however, the severity of the disease and the extent of alveolar bone loss increased with advancing age (Table 4). In this study, the mean alveolar bone loss was found to increase with age, which is in agreement with the findings of Nielsen et al., Papapanou et al., and Kasaj et al.^18-20^

In this study, the mean alveolar bone loss was found to be greater at the distal surfaces of the maxillary and mandibular first molars compared to the mesial surfaces, which is consistent with the findings ofNielsen et al.^18^ However, this result is not in agreement with the studies conducted by Larato, Persson et al., Wouters et al., and Muller et al., which reported more defects on the mesial surfaces than on the distal surfaces.^5,10,21,22^ The greater amount of bone loss in the posterior maxillary and mandibular teeth may be attributed to an increased incidence of interproximal defects^23^ and the presence of more reactive, vascular cancellous bone, as opposed to the less vascular cortical bone.^18,24^

The findings of this study will be helpful in providing baseline information regarding the amount of alveolar bone loss in the maxillary and mandibular permanent first molars among the Nepalese population, as well as the influence of age, gender, frequency of oral hygiene maintenance, and smoking status on ABL. The current study showed a higher amount of alveolar bone loss in patients of increasing age, those who brushed their teeth once a day compared to twice a day, and in smokers compared to non-smokers. These findings may also assist dental practitioners in identifying risk factors associated with alveolar bone loss in periodontitis patients, thereby aiding in treatment planning and prognosis assessment.

However, the findings and subgroup differences should be interpreted with caution, as this is a descriptive study and reflects data from a specific geographic region within the Nepalese population. Therefore, further studies with larger sample sizes across different regions of Nepal are recommended to address these limitations and to validate and generalize the results

## CONCLUSIONS

Within the limitations of the present study, the mean alveolar bone loss was found to be higher in the maxillary permanent first molars, particularly at the distal sites of both maxillary and mandibular permanent first molars. Greater bone loss was also observed among smokers, individuals in older age groups, and those who brushed their teeth only once a day. However, no difference in mean alveolar bone loss was found between males and females.

## Data Availability

The data are available from the corresponding author upon reasonable request.
